# The Biology and Therapeutic Implications of Tumor Dormancy and Reactivation

**DOI:** 10.3389/fonc.2018.00072

**Published:** 2018-03-19

**Authors:** Amit S. Yadav, Poonam R. Pandey, Ramesh Butti, N. N. V. Radharani, Shamayita Roy, Shaileshkumar R. Bhalara, Mahadeo Gorain, Gopal C. Kundu, Dhiraj Kumar

**Affiliations:** ^1^Laboratory of Tumor Biology, Angiogenesis and Nanomedicine Research, National Centre for Cell Science, Pune, India; ^2^Laboratory of Genetics, National Institute on Aging-Intramural Research Program, National Institutes of Health, Baltimore, MD, United States; ^3^Department of Cancer Biology, The University of Texas MD Anderson Cancer Center, Houston, TX, United States

**Keywords:** cancer metastasis, dormancy, reactivation, tumor microenvironment, epithelial to mesenchymal transition

## Abstract

Advancements in the early detection of cancer coupled with improved surgery, radiotherapy, and adjuvant therapy led to substantial increase in patient survival. Nevertheless, cancer metastasis is the leading cause of death in several cancer patients. The majority of these deaths are associated with metastatic relapse kinetics after a variable period of clinical remission. Most of the cancer recurrences are thought to be associated with the reactivation of dormant disseminated tumor cells (DTCs). In this review, we have summarized the cellular and molecular mechanisms related to DTCs and the role of microenvironmental niche. These mechanisms regulate the dormant state and help in the reactivation, which leads to metastatic outgrowth. Identification of novel therapeutic targets to eliminate these dormant tumor cells will be highly useful in controlling the metastatic relapse-related death with several cancers.

## Introduction

Metastasis is a continuous biological process consists of an orderly sequence of basic steps including local invasion, intravasation, extravasation, and colonization. These classical events of metastasis help in understanding the complex array of biological properties that are necessary for the progression of primary malignancy to overt metastasis (1, 2). It involves dissemination of malignant cells from the primary tumor to the distant sites and their proliferation at metastatic sites, which leads to failure of vital organs (1, 2). The kinetics of the metastasis have been highly explored in the past decade. Despite significant research efforts and discoveries made in recent years, the precise reasons for tumor relapse remain largely unknown. There has been significant progress in basic cancer research and clinical oncology; however, metastasis remains to be a key challenge in cancer therapy. Systemic studies on understanding the cellular and molecular mechanisms involved in metastasis might be useful in developing novel diagnostic and therapeutic strategies for metastasis prevention. However, biological, clonal and genetic heterogeneity within or between tumors are the biggest challenges in metastasis research (1, 2). The differential progression of certain cancer subtypes under the distinct selective conditions exists in various tissues leads to metastatic speciation. Disseminated cancer cells might exhibit slow growth in order to adapt to the host microenvironment for the metastatic expansion (3–7). These processes are mirrored by several cancer relapse kinetics in a tissue-specific manner and by the manifestation of distinct organ tropism (3–7). Metastasis might be developed without clinical symptoms after a long period of postsurgery (8). During this period, circulating tumor cells (CTCs) or disseminated tumor cells (DTCs) stay in the dormant state through inhibition of cell proliferation and activation of cell survival pathways (9, 10). The dormant tumor cells remain at low numbers after primary tumor resection. These cells are undetectable for long period and may be the reason for continued asymptomatic residual disease progression and treatment resistance (11–14). However, by understanding more about the biology of dormant cancer cells, the potential treatment strategies can be developed to combat the asymptomatic residual disease. The therapies targeting the mechanism of tissue-specific metastasis might open up new clinical avenues for the management of various cancer progression (15). However, to determine whether dormant solitary cells or micrometastases are valid targets for therapy, the cellular and molecular biology of tumor dormancy and reactivation need to be explored. This review emphasizes on the cancer dormancy, metastatic reactivation and the molecular mechanisms underlying these phenomena.

## Tumor and Metastatic Dormancy

**Figure 1:**
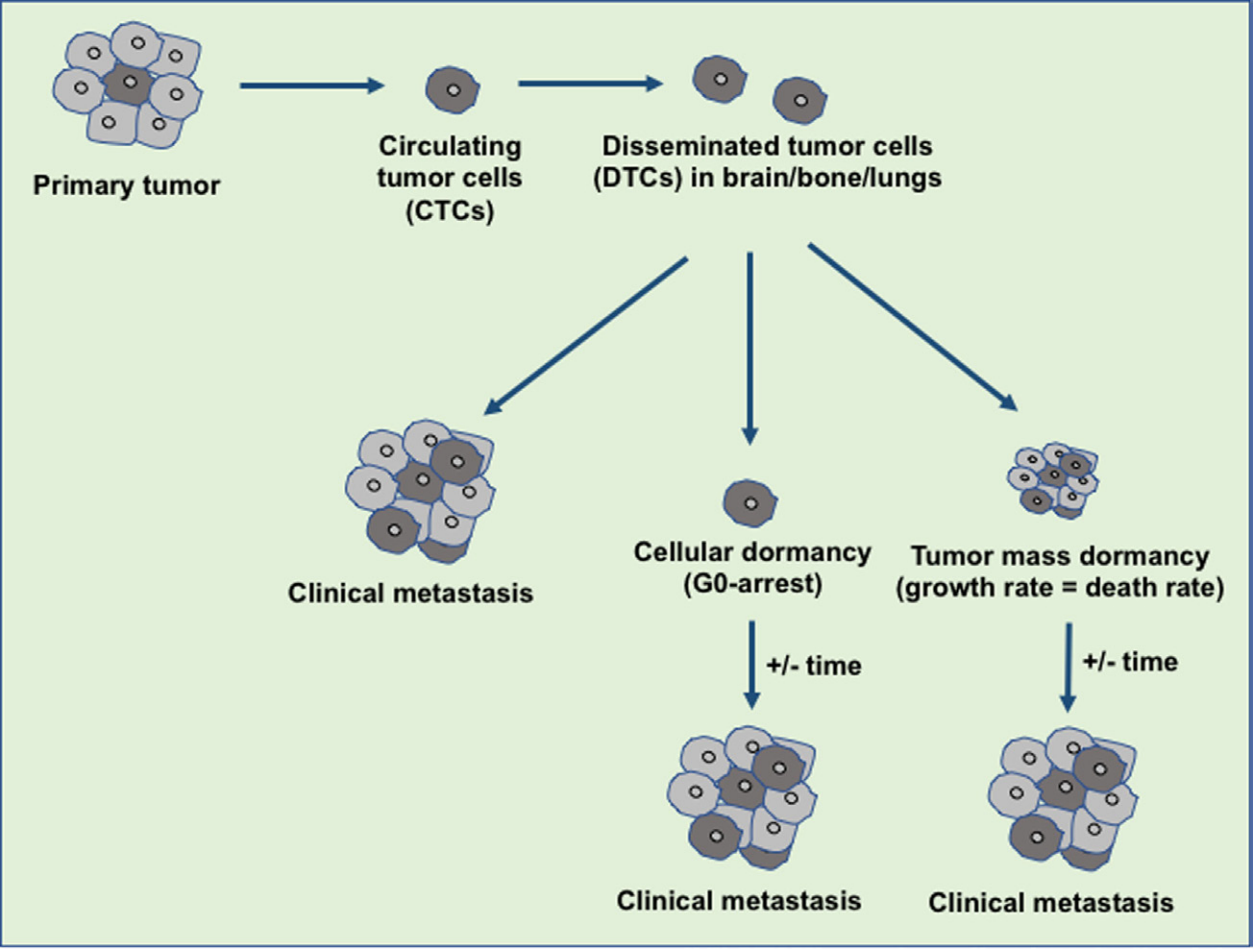
An overview of disseminated tumor cells (DTCs) in dormancy and clinical metastasis relapse. During the metastasis, the disseminated primary tumor cells developed the secondary tumor in the distant organ sites immediately or at a later stage. The tumor microenvironment or the intrinsic factors decide the fate of the DTCs either to develop clinical metastasis or to maintain the dormant state. Over the years, these dormant tumor cells escape from dormancy state and develop the clinical metastasis.

**Figure 2:**
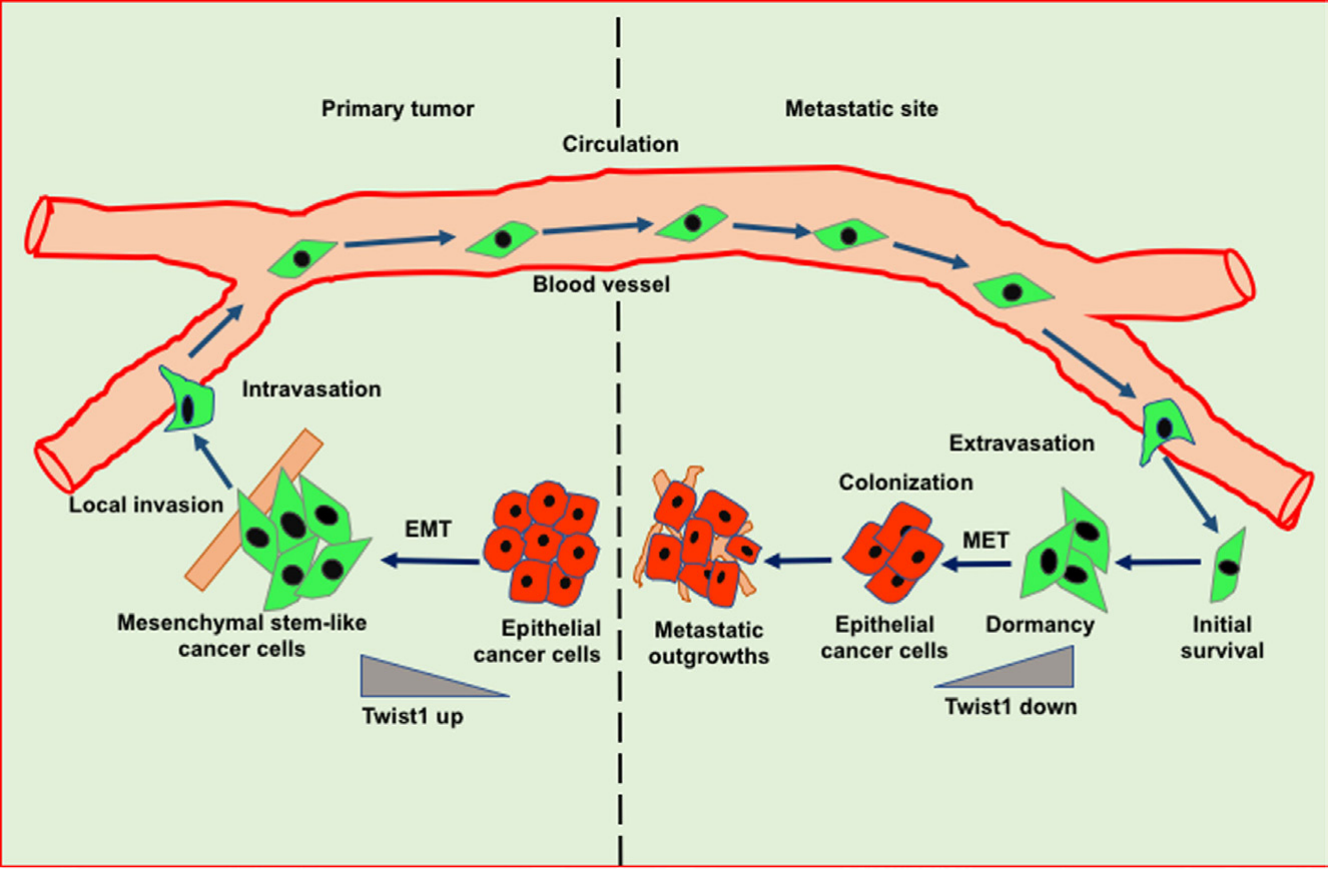
Role of epithelial to mesenchymal transition (EMT) and mesenchymal to epithelial transition (MET) in invasion-metastasis cascade. Cancer cells undergo EMT to acquire stemness and invasion potential leading to cancer cell dissemination. In the target organ, disseminated cancer cells encounter inhibitory signals resulted in the arrest in cell cycle thereby leading to dormancy. Cancer cells undergo MET in order to acquire epithelial features such as proliferation to form metastatic outgrowth in the target organs.

Tumor dormancy is a clinical process that eventually associates with local recurrences or cancer metastases. During this process, the residual disease might be present even after the treatment of primary tumors either in the forms of CTCs, DTCs, and/or micrometastases which have the capability of evading the treatment and survive in a quiescent state. Traditional chemotherapies are most effective on proliferative cells, however, ineffective toward the dormant cells (16). The dormancy nature of the tumor may be reflected by cellular or tumor mass dormancy. In cellular dormancy, cells halt in the G0 phase of the cell cycle and under favorable environmental conditions, they get reactivated by escaping from G0 cell cycle arrest (17). Moreover, during tumor mass dormancy tumor kept constant at a limited size owing to a balance between cell proliferation and cell death. Additionally, angiogenesis and immune response play an important role in maintaining the tumor mass dormancy (18). Dormant cells remain asymptomatic for months, years or even decades and eventually they undergo clinically detectable overt metastatic relapse as shown in Figure 1 (19). Interestingly, dormant cancer cells have also been observed in the primary tumors that undergo epithelial to mesenchymal transition (EMT) to develop migratory and invasive phenotypes (20). In primary tumor dormancy development, somatic mutations play a critical role to withstand apoptosis, senescence, and evade the immune system and trigger neoangiogenesis. In addition, cells undergoing metastatic dormancy are also governed by extracellular matrix (ECM) niches that induce positive signals such as Wnt and Notch and attenuate negative signals like bone morphogenetic protein (BMP) (21). On the contrary, tumor cells at premetastatic sites may undergo dormancy due to delayed adaptation and complex interaction with the local microenvironment (Figure 2).

Several signaling pathways such as RAS-MEK-ERK/MAPK and PI3K-Akt play a crucial role during the process of cancer dormancy (17). Additionally, stress signals, including oxidative response and activation of unfolded protein response (UPR) also have a major contribution to metastatic dormancy and reactivation (21). In addition to various signaling pathways, DNA repair mechanism, and genomic instability also contribute to cancer dormancy (22). It is reported that primary tumor microenvironment may generate a dormant subpopulation, which is capable of evading therapy and responsible for metastatic relapse. It has been shown that metabolic pathway plays a crucial role in dormancy. Studies have demonstrated that altered lipid metabolism coupled with accumulation of reactive oxygen species helps in metastatic recurrence (23).

## Metastatic Niche in Tumor Dormancy and Reactivation

Several reports suggest that cancer cells undergo a protracted period of dormancy by the inhibitory molecular cues derived from primary tumors or restrains caused by target organ microenvironment (24–27). Bidirectional interaction of metastatic tumor cells with microenvironmental niches are imperative for the reactivation of dormant metastatic cells as well as the induction of mesenchymal to epithelial transition (MET) to sculpt the formation of macrometastases. Premetastatic niche provides a favorable microenvironment during metastasis development. Metastatic niche formation includes inflammation, immunosuppression, angiogenesis/vascular permeability, organotropism, lymphangiogenesis, and cellular reprogramming (28). Further, attachment of tumor cells to native basement membrane facilitates their survival, functional differentiation and growth arrest. This suggests that basement membrane is able to contribute to dormancy properties of DTCs. The DTCs often home to the distant organs where the primary basement membrane is mostly vascular in nature such as bone marrow, lung, liver, and brain (29, 30). Studies have shown a close association between DTCs and vascular basement membrane by using the mouse models of breast tumor dissemination (31). Ghajar et al. have shown that endothelial-derived thrombospondin-1 (TSP-1) induces the quiescence in breast cancer cells and this suppressive cue lost during neovasculature. The time-lapse analysis showed that sprouting vessels permit and accelerate breast cancer cell outgrowth (31). Further, they have shown that recreation of the organotypic microvascular niche of lung and bone marrow promotes dormancy and quiescence (31). It has also been shown that attachment with microvasculature in the perivascular niche is necessary for DTCs survival in mouse brain (32). These data support that endothelial cells help in dormancy induction whereas neovascularization in perivascular niche supports reactivation of dormant cells that leads to metastatic outgrowth.

A recent report suggests that a subset of macrophages (TAMs), known as metastasis-associated macrophages (MAMs), are enriched in metastatic breast cancer as compared to primary tumors. Flt1-regulated signaling in these MAMs upregulates inflammatory gene signature which is imperative for cancer cell survival during metastatic seeding (33). In addition to TAMs, circulating VEGFR1^+^ and bone marrow-derived CD11b^+^Gr1^+^ myeloid cells are involved in premetastatic niche formation (34–36). Myeloid cells expressed versican, an ECM proteoglycan, plays a key role in inducing proliferation of cancer cells to form metastatic outgrowth in the lung (35). CYP4A-induced TAMs promote premetastatic niche formation and metastasis in the lung by recruitment of VEGFR1^+^ myeloid cells (36). Moreover, induction of TGF-β in myeloid cells by natural killer T cell-derived interleukin (IL)-3 suppresses immune responses and controls tumor recurrence (37). Other stromal cells such as fibroblasts and endothelial cells present at premetastatic niche also play an important role in this phenomenon. Cancer-associated fibroblasts show activated phenotype and are integral components of premetastatic niche. Studies show that breast cancer metastasis-associated fibroblasts secrete higher level of IL-6 that promotes malignant growth (38). Furthermore, systemic factors derived from primary tumors induce the fibronectin synthesis by fibroblasts to form premetastatic niche by recruiting a fibronectin-binding integrin α4β1^+^ hematopoietic progenitor cells. These hematopoietic progenitor cells remodel the local microenvironment by producing MMP-9 and other factors and stimulating angiogenesis (34, 39, 40). Hence, the metastatic niche plays a pivotal role in the survival, maintenance, and reactivation of DTCs.

## MET and Metastatic Reactivation

Epithelial to mesenchymal transition-driven mesenchymal features in cancer cells enable them to invade and metastasize to the distant organs. Several studies suggest that EMT-inducing transcription factors such as Twist and Snail show inhibitory effects on cancer cell proliferation, however, these factors induce migratory potential by downregulating the cadherin junctions (41). A reverse phenomenon of EMT known as MET helps in the tumor relapse or dormancy reactivation through the restoration of epithelial features. Interestingly, during MET tumor cells actively proliferate and regain adhesive junctions to communicate with the surrounding niche of the metastatic sites (Figure 2) (42). Recent reports have shown that blockade of the TGFβ/Smad2 pathway by versican promotes MET phenotype (43). Induction of MET in breast cancer cells is associated with increased metastatic colonization. Tsai et al. have found that attenuation of Twist1 expression promotes the metastatic outgrowth by inducing MET and proliferation of cancer cells (44). Additionally, Prrx1 another EMT transcription factor confers the migratory and invasive properties of cancer cells. Various studies showed that loss of Prrx1 contributes to metastatic colonization by stimulating MET phenotype. Moreover, downregulation of Prrx1 is associated with metastatic disease and poor survival of patients (45). Several studies showed that accumulation of genetic and epigenetic changes in tumor cells facilitated them to revert dormancy and undergo metastatic reactivation. Posttranslational modification of histones is extensively studied epigenetic change which has been observed in transcriptional activation of various EMT/MET-associated genes. The recent report suggests that H3K27me3-demethylase KDM6A expression toggles during EMT and MET processes. KDM6A catalytically removes di- and tri-methyl groups from H3K27me3 suppressive mark of the H3K4me3/H3K27me3 bivalent promoters; to promote the expression of target genes associated with differentiation, proliferation and cellular adhesion (46). Collectively, these studies suggest that the stromal cell signaling and MET contribute to metastatic reactivation.

## Mechanisms of Cellular Dormancy

Metastatic dormancy is a result of growth arrest either in a single DTC termed as cellular dormancy or in micrometastatic lesions called as tumor mass dormancy. Cellular dormancy marked by a quiescent state in DTCs is associated with the decline in Ki67 expression, a proliferation marker or G0/G1 cell cycle arrest. There are various cellular and molecular mechanisms through which DTCs undergo dormancy which is discussed below.

### Stress-Induced Signaling and UPR in Cellular Dormancy

**Figure 3:**
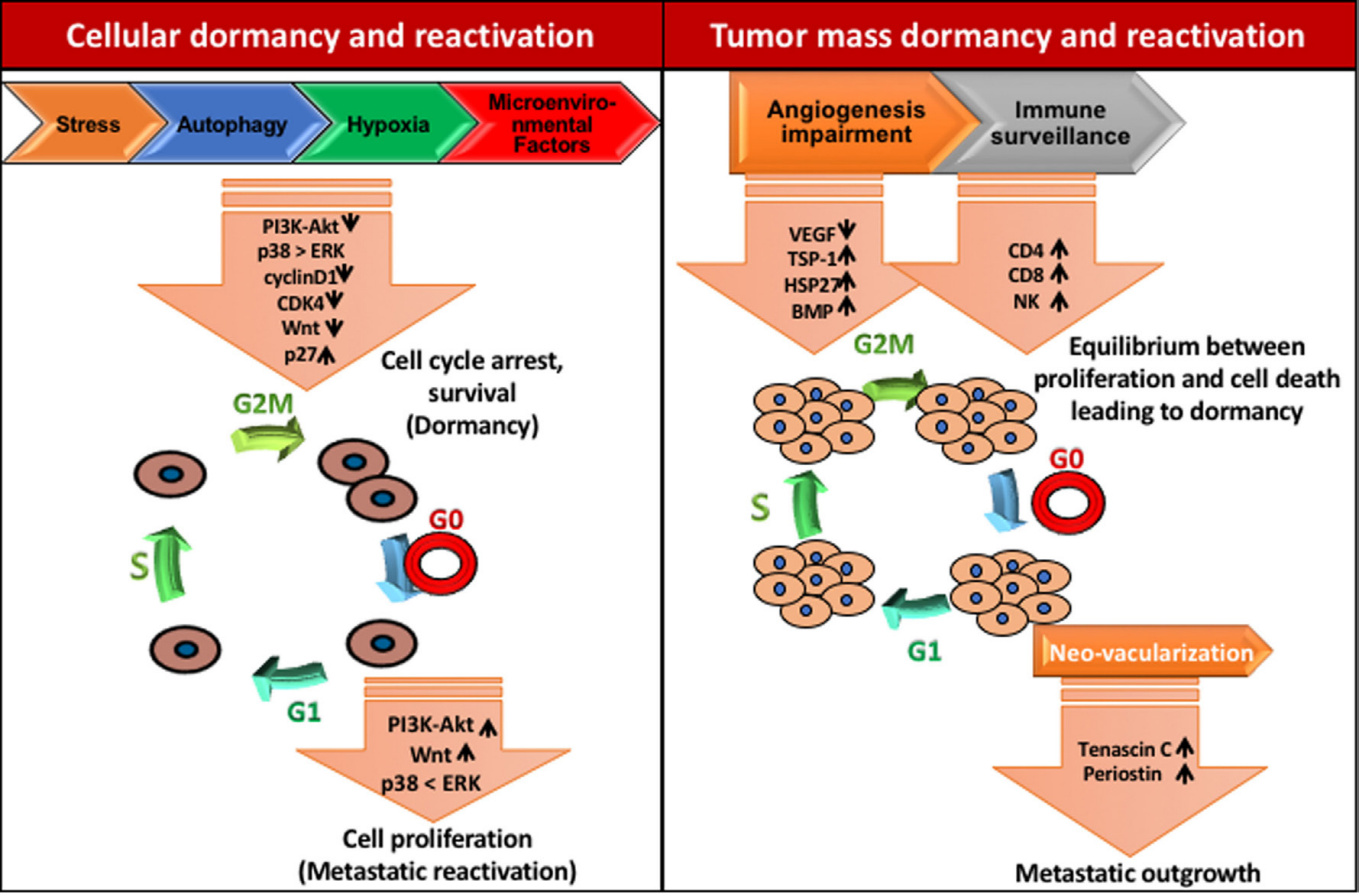
Mechanisms of tumor dormancy. Solitary cell dormancy (cellular dormancy, left) is caused by cell cycle arrest and induction of survival mediated by various signaling cascades including downregulation of PI3K-Akt, ERK, and Wnt signaling and upregulation of p38 MAPK signaling. Tumor mass dormancy (right) is a result of the balance between proliferation and cell death due to less blood supply and immune surveillance.

Mitogen, stress signal, and other factors present in the premetastatic niche may be responsible for cell cycle arrest and dormancy. Crosstalk between mitogen and stress-induced signaling pathways are crucial for cellular dormancy. Studies have shown that a set of genes selectively affects the growth at the secondary site including MKK4, MKK6, and Nm23-H1. Interestingly, MKK4 and MKK6 are upstream activators of p38 while, Nm23-H1 indirectly downregulates ERK1/2 by inhibiting EDG2 LPA receptor, a strong activator of ERK1/2 (47). Hence, ERK/p38 signaling ratio seems to have a crucial role in cancer cell dormancy and reactivation (Figure 3). Several studies showed that the enhanced levels of p38 MAPK over ERK1/2 upon downregulation of uPAR induces dormancy in squamous cell carcinoma (48, 49). Researchers have demonstrated that Minibrain-related kinase/dual specificity tyrosine phosphorylation-regulated kinase 1B (Mirk/DYRK1B) blocks cyclin D1 and CDK4 which further regulates the survival signals and cell cycle arrest in pancreatic and ovarian cancer cells (50–52). Likewise, MAPKK4 has been shown to exert dormancy by the upregulation of JNK pathway in prostate and ovarian cancer cells (53, 54).

Several reports have shown that the upregulation of various UPR-associated genes like Grp78, Grp94, PDI, heat shock protein 47 (HSP47), and cyclophilin B in dormant cells play a crucial role in metastatic dormancy (55–58). Ranganathan et al. showed that stress-induced p38 activation leads to upregulation of the endoplasmic reticulum (ER) chaperone BiP. Further, this factor increases the activation of the ER stress-activated PERK signaling that results in higher survival and therapy resistance in dormant cells (56). Additionally, p38 kinase-mediated activation UPR also induces the expression of the ER stress-regulated transcription factor ATF6 and promotes mTOR-mediated survival of the dormant cells (59). Moreover, the mechanistic analysis in DTCs derived from bone marrow of breast cancer patient revealed that the expression of Grp78, a UPR protein, upregulated in low oxygen and glucose conditions and promotes higher proliferation and sustained survival (58).

Microenvironmental factors like BMPs and growth arrest-specific 6 (GAS6) derived from mesenchymal cells and osteoclasts, respectively, can curb proliferation and induce dormancy in cancer cells (Figure 3). By using the prostate cancer bone metastasis model, Kobayashi et al. have demonstrated that BMP7 promotes dormancy. BMP7 induces the expression of metastasis suppressor gene N-myc, leading to the activation of p38 MAPK, p21, and cell cycle arrest (60). Moreover, Shiozawa et al. reported that the activation of the GAS6 receptor in prostate cancer cells in the bone marrow environment plays a critical role in establishing metastatic tumor cell dormancy (61). The recent study has demonstrated that latency competent cancer cells from early-stage human lung and breast carcinoma cells can self-imposed in a dormant stage by downregulating Wnt signaling and inducing Sox-dependent stem-like state (62). Altogether, these results suggest that stress signaling helps in the single cell dormancy by arresting proliferation and enhancing survival of DTCs in the premetastatic niche.

### Hypoxia and Dormancy

In the tumor microenvironment, hypoxia plays a critical role during tumor development and metastasis. Fluegan et al. have explored the influence of hypoxia on the fate of DTCs. They report that hypoxia enhances the expression of key dormancy genes like NR2F1, DEC2, p27 in head and neck squamous cell carcinoma (HNSCC) and primary breast tumor. Posthypoxic solitary DTCs in patient-derived xenografts and transgenic mice show NR2F1^hi^/DEC2^hi^/p27^hi^/TGFβ2^hi^ population with dormant phenotype. NR2F1 and HIF1α involved in the regulation of p27 expression in posthypoxic dormant DTCs. Moreover, hormone receptor-dependent breast cancer cells exhibit higher affinity toward NR2F1-dependent dormancy (63). Harper et al. have delineated the molecular mechanism by which HER2 aberrantly activates a program for early dissemination and generation of DTCs. These early DTCs exhibit p-p38^lo^p-Atf2^lo^Twist1^hi^E-cad^lo^ expression pattern and an EMT-like dissemination program without complete loss of epithelial feature which was recovered after inhibition of HER2 and Wnt signaling (Figure 3). Interestingly, the dormancy feature in these early DTCs was p38-independent and even after being Twist1^hi^E-cad^lo^ and dormant, they were able to initiate metastasis (64). These data indicate that the development of dormancy feature is governed by several intrinsic and extrinsic programs and by contextual cues.

### miRNAs in Cellular Dormancy

miRNAs play an important role in the various biological process. It has been shown that miRNAs may affect the hallmarks of cancer, including sustained proliferation, blocking growth inhibition signals, resisting cell death, inducing invasion, metastasis, and angiogenesis (65). Ono et al. have described the role of miRNA derived from bone marrow mesenchymal stem cells in the induction of dormancy in metastatic breast cancer cells isolated from bone marrow of the mice. This study showed that higher expression of miR-23b in metastatic breast cancer cells leads to dormant phenotype by downregulation of MARCKS gene, associated with cell cycle progression and motility (66). The data also showed a consensus set of 19 miRs with the potential role in governing the phenotypic switch of human dormant breast carcinoma, glioblastoma, osteosarcoma, and liposarcoma to outgrowth. They have shown that loss of dormancy-associated miRs (DmiRs, 16/19) reactivate the fast growth of the dormant tumors. However, reestablishment of a single DmiR (miR-580, 588, or 190) results in the phenotypic switch of fast-growing angiogenic tumor toward prolonged dormancy (67).

### Autophagy and Dormancy

Autophagy is an extremely conserved self-degradation process, which has an important role in cancer stem cells (CSCs) regulation and tumor cell survival. Several reports suggest that DTCs possess CSCs properties, which prompted researchers to explore the potential role of autophagy in cancer cell dormancy and stress response. Various groups have shown that autophagy helps in the survival of DTCs for protracted periods (68, 69). Autophagy supports DTCs survival by sustaining amino acid levels, ATP production and blocking energetic catastrophe (69–71). Further, induction of autophagy has linked to dormancy. Liang et al. have shown that the activation of LKB1-AMPK leads to induction of ULK1, which initiates autophagy. Further, this pathway activates p27kip1-dependent growth arrest (G1 arrest) and downregulation of this signaling induces apoptotic cell death (72). They have proposed a novel mechanism which links autophagy stimulation, growth arrest and apoptosis. In recent finding, Lu et al. have shown the role of tumor suppressor protein, aplasia Ras homolog member I (ARH1) which partly induces autophagy by inhibiting PI3K/Akt pathway. This study shows that reexpression of ARH1 in SKOv3 ovarian cancer xenograft results in tumor regression likely due to autophagy. However, the xenograft exhibited prolonged growth arrest indicating the onset of dormancy which was reversed after subsequent knockdown of ARH1 (73). These studies link the onset of autophagy with growth arrest/quiescence program and survival which proposing a key role of autophagy in dormancy.

## Mechanisms of Tumor Mass Dormancy

In contrast to single cell dormancy, tumor mass dormancy is governed by a balance between the rate of proliferation and apoptosis in micrometastatic lesions. The tumor mass dormancy is induced by slow proliferation, restrained blood supply and active immune response. Recent studies reveal that the frequency of osteolytic bone metastasis depends on metastatic niche environment rather than the number of cancer cells (74, 75). Moreover, stromal factors such as TGFβ and BMPs have potential role in the regulation of tumor initiation, proliferation and maintenance of the quiescent state. Bragado et al. have suggested that TGFβ2 induces slow cycling and quiescence in cells by suppressing CDK4 and inducing p27 in HNSCC (76). Interestingly, it has been shown in multiple myeloma that a small population of Ki67^+^ cells coexists with dormant cells, proposing that for the reactivation defined niches are essential (77, 78). Unfortunately, the mechanisms behind long-term metastatic dormancy are highly unexplored. However, sustenance of tumor mass dormancy relies on the cellular mechanisms that induce slow cycling.

Micrometastatic lesions require the higher blood supply to grow beyond 1–2 mm, which leads to the induction of vessel formation by secretion of angiogenic factors like VEGF (79). Therefore, the antiangiogenic signaling mechanisms could be an interesting factor, which maintains the tumor mass dormancy (31, 80). These studies show that upregulation of TSP-1, an angiogenic inhibitor induces poor vascularization and dormancy in breast cancer, glioblastoma, osteosarcoma, and liposarcoma under *in vivo* conditions (81). Chaperons like HSP27 also regulate the angiogenesis and dormancy. Ablation of HSP27 in breast cancer prompts the long-term *in vivo* dormancy while its upregulation results in dormancy exit and enhanced vascular density (80).

Clearance of tumor cells by immune system contributes to another mechanism of tumor mass dormancy. Cancer cells coevolve in a microenvironment where the immune system is suppressed. However, DTCs do not have such support and eventually, most of these cells die due to the natural immune response. It has also been reported that immune system regulates the number of DTCs as well as the size of micrometastatic lesions (82). Additionally, the presence of DTCs in bone marrow of breast cancer patients showed the correlation with the higher immune cell subpopulations including NK cells, macrophages and T lymphocytes. All these cell types are known to be involved in rejection of primary tumors and metastasis, which leads to tumor dormancy (83).

## Mechanisms of Metastatic Reactivation

Dormant cancer cells may be subjected to reactivation to initiate metastasis in response to specific signals from their specialized niche, which maintains the balance between the self-renewal and production of differentiated progeny (84–88). Cancer cells start preconditioning the host microenvironment even before seeding by secreting various soluble factors (39, 89). Heparanase, osteopontin, and lysyl oxidase facilitate the invasion, survival, and proliferation of metastatic breast cancer cells (90–92). After extravasation, DTCs may encounter different niches including perivascular niche. It has been shown that attachment of DTCs on the abluminal surface of mature blood vessels promotes dormancy through endothelium-derived TSP-1, while neovascularization creates a local microenvironment favoring metastatic reactivation. After neovascular sprouting, vessel homeostasis gets disrupted and endothelial cells start secreting tumor-promoting signals and growth factors like ECM proteins, periostin and active TGFβ that leads to micrometastatic outgrowth (31). It has been reported that ECM protein tenascin C activates Notch and Wnt signaling leading to enhanced metastatic outgrowth (93, 94). TGFβ helps in the production of periostin from stromal fibroblasts and endothelial cells in the neovascular area that supports metastatic outgrowth (31, 95). Further, Gao et al. have reported that Coco a secreted antagonist of TGFβ ligand reactivates solitary breast cancer cells at organ-specific metastatic sites by shielding metastasis-initiating cells from inhibitory signals provided by lung-derived BMP proteins. A large group of patients expressing Coco showed predicted relapse to lung but not to brain and bone due to the absence of bioactive BMP (96). Hence, the metastasis-initiating cells may promote the permissive niche comprising of matrix proteins which are involved in activation of specific signaling pathways such as Wnt and Notch that in turn activate their self-renewal. Recent report suggests that the TAM family of receptor tyrosine kinases TYRO3, AXL, and MERTK have a potential role in dormancy regulation in prostate cancer. MERTK stimulates the reactivation of dormant prostate cancer cells through MAP kinase-dependent mechanism, which involves p27, pluripotency transcription factors, and histone methylation (97).

## Therapeutic Implications of Dormancy and Reactivation

**Figure 4:**
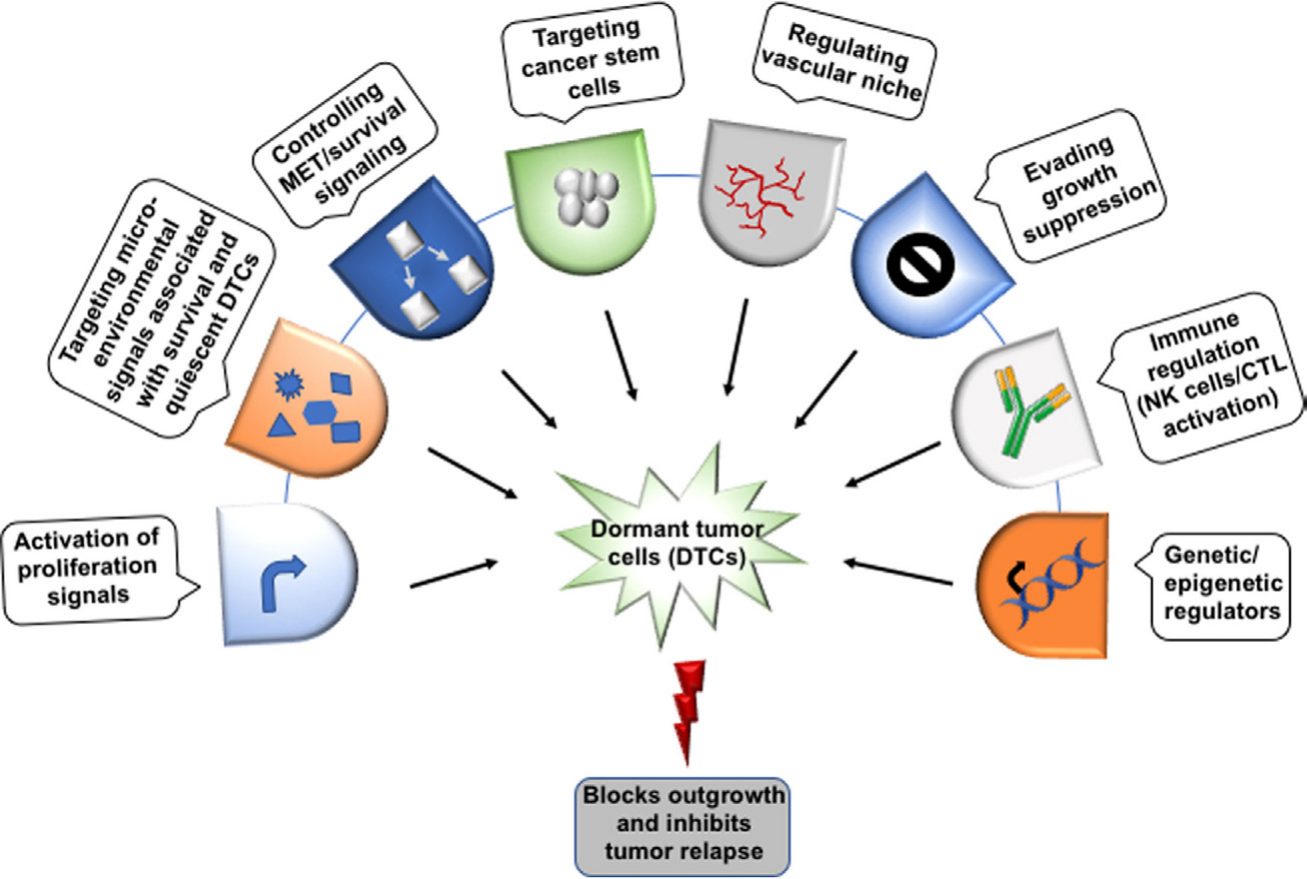
Therapeutic implications of dormant tumor cells. The possible target sites to eliminate the dormant tumor cells (DTCs) in order to regulate metastatic relapse. Though the direct evidence to target the dormant cells yet needs to identify extensively. Moreover, the dormant cells can be targeted at several checkpoints including epigenetic regulators (DNMT1, EZH2), immune cells (NK cells/CTL) activation, evading growth suppression, vascular niche, quiescent cancer stem cells, survival signaling, and the microenvironment signals (bone morphogenetic protein 4/7, CXCL12, TRAIL, growth arrest-specific 6, TGFβ-2, BME, and thrombospondin 1) that help in the maintenance of the dormant state.

Recent achievements in cancer therapy and increased overall survival motivate the researchers to look for new diagnostics for the patients at high risk of late metastasis and therapeutic system targeting DTCs. The limitation of current conventional and adjuvant therapies to prevent relapse, is they basically target growing tumor cells rather than DTCs. The systemic nature of the metastatic disease along with the heterogeneity of metastatic tumors, complex inter-connected pathways and the resistance against therapy makes its pharmacological management very difficult. Hence, there is a need to focus on preventing metastasis (98, 99). Bone-modifying drugs have been used clinically for management of bone metastasis-related morbidity. However, when they used in the preventive adjuvant setting against cancer, inconclusive results were observed (98–100). A detailed understanding of the mechanism of metastatic dormancy and colonization along with innovative therapeutics must be developed to solve this medical dilemma. For this, therapeutic agents that can inhibit metastasis by targeting metastatic cell-autonomous functions and mechanisms responsible for dormancy and their survival would serve as a new opportunity to prevent minimal residual disease (Figure 4). Since DTCs are highly dependent on signaling, hence targeting these pathways may be helpful in enhancing the efficacy of adjuvant therapy and managing the metastatic relapse. Based on existing reports, targeting Src, Akt, or Tor by using their inhibitors alone or in combination with chemotherapy can be a potential approach for the treatment of minimal residual disease. Studies under *in vivo* preclinical and 3D *in vitro* model of dormancy demonstrated that targeting the Src family kinase and MEK1/2 using their specific inhibitors resulted in apoptosis in a large fraction of the dormant cells and delayed metastatic outgrowth in breast cancer (101). Inhibition of Src kinase family signaling or Src knockdown leads to the nuclear localization of cyclin-dependent kinase inhibitor p27 resulting in prevention of metastatic outgrowth; however, it did not affect the survival of the dormant cells. MEK1/2 inhibitors that block the downstream ERK1/2 signaling suppresses DTCs survival. Several studies have shown that the various phenotypic and functional similarities are shared between metastasis-initiating cells and CSCs. Hence, CSCs targeted therapies may be effective in the treatment of metastatic disease. Moreover, stem cell signaling pathways also induce resistance to chemotherapy. Thus, combination therapy targeting stem cell pathways like Notch and Wnt along with canonical oncogenic pathway or reactivating BMP signaling may be effective in metastatic disease therapy. It has been shown that autophagy promotes the survival of the dormant cancer cells. Interestingly, inhibition of autophagy reduces clonogenic survival of lung, cervical, and breast cancer cell (102). Therefore, autophagy can also be considered as a therapeutic target in cancer metastasis.

Immunotherapy is being explored extensively for cancer management. Saudemont et al. have shown NK cells-based immune therapy targets dormant cells. Their study demonstrated that NK cells activated by CXCL10 can kill dormant tumor cells which are able to resist CTL-mediated lysis (103). As discussed earlier, secretory molecules and cytokines in microenvironment also play a key role in the regulation of dormancy (Figure 4). Osteopontin, an ECM protein has been reported in the progression of various cancers (91, 104, 105). Boyerinas et al. have shown that stromal osteopontin helps in anchoring leukemia cells in bone marrow premetastatic niche and support dormancy by inducing cell cycle arrest. Neutralizing the osteopontin resulted in the proliferation of dormant cells and enable them sensitive to chemotherapy (106). Hence, the better understanding of the mechanism governing dormancy and reactivation and the role of metastatic niche may help in the identification of new potential therapeutic targets for the treatment of minimal residual disease.

## Future Direction

Tumor dormancy and reactivation has become an interesting point as a key element of tumor evolution and metastatic relapse. Although metastasis-initiating cells undergo dormancy and ultimately get reactivated under the influence of microenvironment signals, various key questions are still unanswered. It will be interesting to explore the phenotypic and functional similarities between DTCs and CSCs, the role of MET, microenvironmental niches and genetic and epigenetic changes in metastasis-initiating cells in metastatic reactivation. Current approaches and models to investigate the molecular basis of metastasis have been very successful. Nevertheless, new approaches need to be discovered in order to gain an in-depth understanding of tumor dormancy and reactivation. Lineage-tracing studies utilizing newly developed reporter systems can provide critical understanding in this area by using the transgenic mouse models which mimic the natural conditions. Moreover, recently invented genetic screening strategy can be useful in quick identification of mediators involved in dormancy and reactivation. Future studies need to be conducted to assess the efficacy of screening the shRNA libraries for the recognition of regulators of dormancy and their potential use in various tumor types and clinical samples.

Advance strategies for characterization of various aspects of CTCs and better access to samples of metastases will be required to complete this goal. With the current progress in the field of metastasis, these questions will be addressed rapidly by designing and implementing the improved strategies for cancer treatment.

## Author Contributions

DK, AY, PP, and RB drafted the manuscript, composed the figures, and critically revised the manuscript. DK and GK conceived the manuscript and finalized the draft. NR, SR, SB, and MG have written some part and revised the manuscript. All authors read and approved the final manuscript.

## Conflict of Interest Statement

The authors declare that the research was conducted in the absence of any commercial or financial relationships that could be construed as a potential conflict of interest.
